# Sequence analyses of a lipoprotein conserved with bacterial actins responsible for swimming motility of wall-less helical *Spiroplasma*

**DOI:** 10.17912/micropub.biology.000713

**Published:** 2023-03-15

**Authors:** Daichi Takahashi, Makoto Miyata

**Affiliations:** 1 Graduate School of Science, Osaka Metropolitan University, Osaka, Japan.; 2 The OMU Advanced Research Center for Natural Science and Technology, Osaka Metropolitan University, Osaka, Japan.

## Abstract

*Spiroplasma* is a genus of pathogenic or commensal cell-wall-deficient helical bacterium. *Spiroplasma* -specific protein fibril and five classes of bacterial actins, MreB1–5, are involved in a helical ribbon structure responsible for helical-cell morphology and swimming motility. A gene for a hypothetical protein—SPE_1229, 7th protein—has been found in the locus coding *mreB* s. In this study, we characterized the 7th protein using *in silico* methods and found that it could be a lipoprotein whose gene is encoded downstream of *mreB3* and conserved in a clade of *Spiroplasma* .

**Figure 1. Characterization of the 7th protein.**
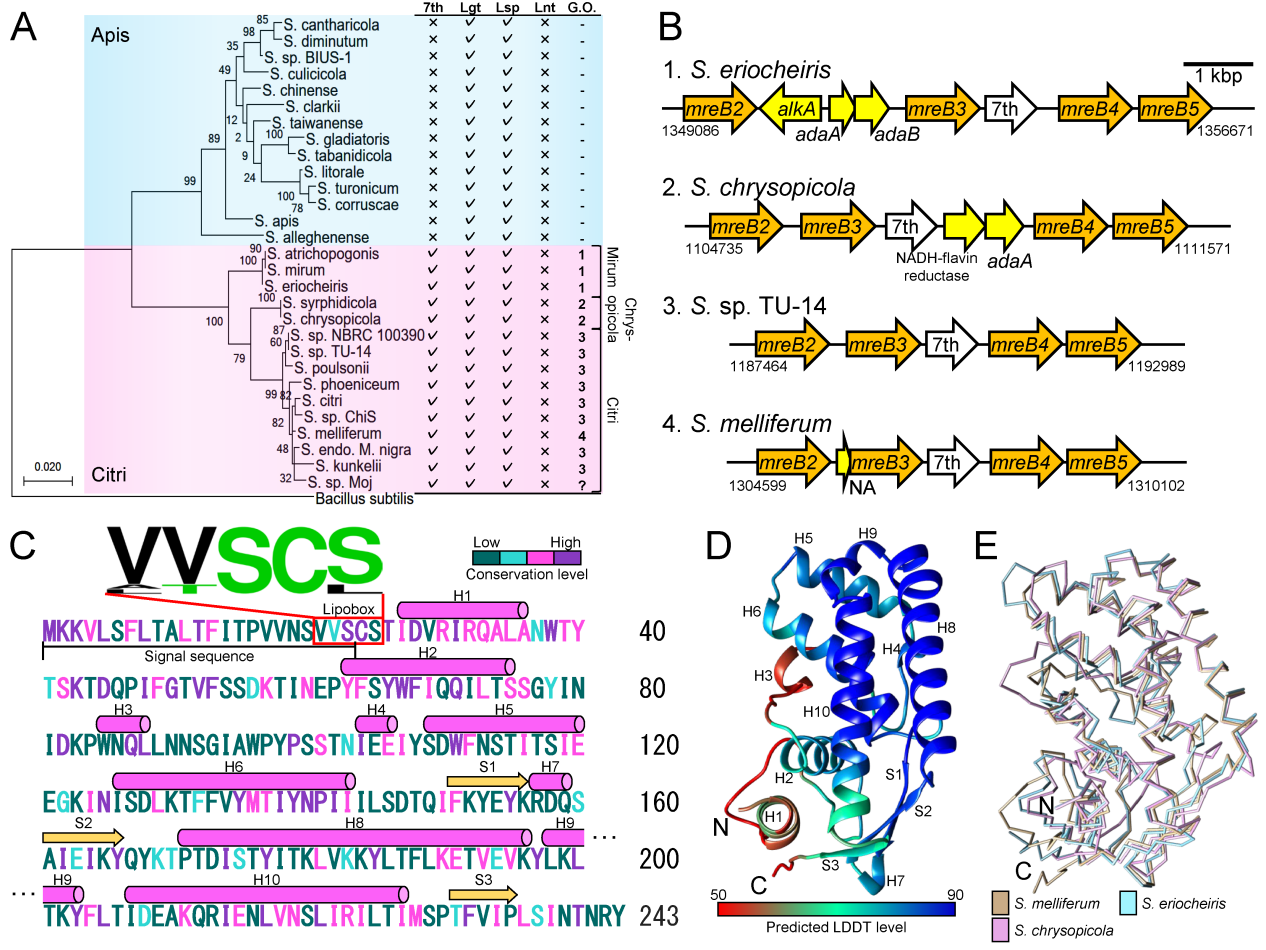
( **A** ) (Left) Maximum likelihood phylogeny of 16S rRNA of *Spiroplasma* . *Bacillus subtilis* 16S rRNA sequence is used as the outgroup. Species belonging to Apis and Citri clades are highlighted using cyan and magenta boxes, respectively. Bootstrap support values were estimated from 500 alignment samples and indicated on each node. The scale bar is in units of the nucleotide substitution numbers per site. (Right) List summarizing the presence of genes for the 7th protein, Lgt, Lsp, and Lnt. The check and cross symbols indicate whether the corresponding gene is present or absent, respectively, on the genome. Column G.O. indicates the patterns of the gene orders from *mreB2* to *mreB5* shown in panel B. Gene organization pattern of *Spiroplasma* sp. Moj could not be determined owing to the insufficient length of the reported contig sequence including the *7th* *gene* , denoted using “?” in the G.O. column. Three classifications in Citri clade (Mirum, Chrysopicola, and Citri) are indicated on the right of the list. ( **B** ) Four patterns of gene orders from *mreB2* to *mreB5* found in Citri clade species. The nucleotide numbers of the *mreB2* initiation and *mreB5* termination points on the genomes are indicated below the corresponding sites. ( **C** ) Amino acid sequence of the 7th protein of *S. melliferum* . Each amino acid is colored based on the four conservation levels (purple for identical, magenta for strongly similar, cyan for weakly similar, and green for diverse) estimated by the multiple alignment of 14 amino acid sequences of the 7th protein. The signal sequence region is indicated using a solid line. The lipobox region is indicated using a red box with its WebLogo on the sequence. The regions for the α-helix and β-strand predicted by AlphaFold2 are indicated using a magenta cylinder and yellow arrow, respectively. ( **D** ) Ribbon representation of *S. melliferum* 7th protein structure predicted by AlphaFold2. The first 24 amino acids at the N-terminus including the signal sequence and lipobox are excluded from the model. Each amino acid is colored by the predicted pre-residue confidence score (LDDT). ( **E** ) Structural comparison among the 7th proteins of *S. melliferum* (beige), *S. eriocheiris* (cyan), and *S. chrysopicola* (magenta).

## Description

*Spiroplasma* belongs to the class Mollicutes. *Spiroplasma* are pathogenic or commensal to plants and arthropods and possess a wall-less helical cell morphology (Cole *et al.,* 1973; Daniels *et al.,* 1973; Paredes *et al.,* 2015; Tsai *et al.,* 2018). They show characteristic swimming motility, in which cells are driven by the continuous switching of cell helicities (Shaevitz *et al.,* 2005; Wada and Netz 2009). Compared to the conventional types of bacterial motility such as flagellar and pili motilities, the swimming force of *Spiroplasma* is generated by conformational changes in an intracellular ribbon structure composed of fibril, a cytoskeletal protein specific to *Spiroplasma* , and bacterial actin MreB (Kürner *et al.,* 2005; Trachtenberg *et al.,* 2008; Liu *et al.,* 2017; Miyata *et al.,* 2020; Sasajima *et al.,* 2022). *Spiroplasma* possesses five classes of MreBs (MreB1–5), which are divided into three functional groups based on sequence similarity: MreB1&4, MreB2&5, and MreB3. Previous experiments on *Spiroplasma* MreB expression in *Escherichia coli* cells and *in vitro* polymerization suggested the following roles of these MreBs: MreB1 and/or MreB4 form a static backbone interacting with fibril filaments; MreB2 and/or MreB5 change the ribbon forms by polymerization dynamics, and MreB3 forms static filaments to anchor MreB1 and/or MreB4 onto the cell membrane (Masson *et al.,* 2021; Takahashi *et al.,* 2022). Previous studies showed that these genes are involved in the swimming motility (Harne *et al.,* 2020a; Harne *et al.,* 2020b; Kiyama *et al.,* 2022; Lartigue *et al.,* 2022). The five classes of MreBs and fibril are conserved in the *Spiroplasma* genus (Ku *et al.,* 2014; Takahashi *et al.,* 2020), suggesting that the swimming mechanism is conserved in *Spiroplasma* , although previous studies on swimming in this genus have focused mostly on four species ( *S. melliferum* , *S. citri* , *S. eriocheiris* , and *S. poulsonii* ) (Shaevitz *et al.,* 2005; Wada and Netz 2009; Liu *et al.,* 2017; Boudet *et al.,* 2018; Harne *et al.,* 2020a; Nakane *et al.,* 2020; Masson *et al.,* 2021; Kiyama *et al.,* 2022; Lartigue *et al.,* 2022; Sasajima *et al.,* 2022). The hypothetical gene *SPE_1229* was found to be encoded by a locus encoding *mreB* s. Although poorly characterized, SPE_1229 and its homologs are found in many *Spiroplasma* species with a corresponding position (Harne *et al.,* 2020a; Kiyama *et al.,* 2022). In this study, we analyzed SPE_1229 using *in silico* methods and in the following sections, we have tentatively named SPE_1229 and its gene as “7th protein” and “ *7th gene* ,” respectively, because the gene position is adjacent to one of the “six” cytoskeletal proteins responsible for *Spiroplasma* swimming.

We first performed a BLAST search to collect amino acid sequences of the 7th protein (Extended Data 1) and searched for its distribution (Figure 1A). Homologs from species other than *Spiroplasma* were not found, suggesting that the 7th protein is specific to *Spiroplasma* . The *Spiroplasma* genus is divided into two clades based on phylogenetic relationships: Apis and Citri-Chrysopicola-Mirum (Citri) (Ku *et al.,* 2014; Paredes *et al.,* 2015; Tsai *et al.,* 2018; Takahashi *et al.,* 2020). The 7th protein is conserved in the Citri clade but not in the Apis clade. Later, we examined the order of genes coding for the 7th protein and the cytoskeletal proteins related to *Spiroplasma* swimming, that is, fibril and the five classes of MreBs. The *7th gene* is always found in a locus that includes *mreB2* , *mreB3* , *mreB4* , and *mreB5* . The gene order from *mreB2* to *mreB5* was classified into four patterns (Figure 1B). In the genomes of 8 out of 10 species in the same clade as *S. citri* (species labeled as Citri in Figure 1A), *mreB2* , *mreB3* , *7th gene* , *mreB4* , and *mreB5* were tandemly encoded in the order shown (Figure 1B-3). The *S. melliferum* genome encoded another hypothetical gene immediately before *mreB3* (Figure 1B-4). In the genomes of three species ( *S. eriocheiris* , *S. mirum* , and *S. atrichopogonis* ), *alkA* (at the minus strand), *adaA* , and *adaB* all of which are responsible for demethylations of DNA (Sedgwick and Lindahl 2002) are encoded between *mreB2* and *mreB3* (Figure 1B-1). *S. chrysopicola* and *S. syrphidicola* encode a gene for NADH-flavin deaminase and *adaA* between the *7th gene* and *mreB4* (Figure 1B-2). Although there is diversity in gene orders at the loci, the *7th gene* is always encoded downstream of *mreB3* (Figure 1B).

To characterize the 7th protein, its amino acid sequence was analyzed using Phobius, a prediction tool for membrane proteins (Käll *et al.,* 2004). All the 7th protein sequences were projected to possess signal sequences for extracellular secretion on the N-terminal 18–23 amino acid residues (Figure 1C, Extended Data 1). At the 21st to 25th position, around the end region of the signal sequence, the [V/L]-[V/T]-S-C-[S/L] motif was conserved in 13 out of 14 sequences of the 7th protein (Figure 1C red box). This motif matches the consensus sequence of the lipobox ([L/V/I]-[A/S/T/V/I]-[G/A/S]-C-X) conserved at the end of a signal sequence in bacterial prolipoproteins (Kovacs-Simon *et al.,* 2011). The structure of the 7th protein predicted by AlphaFold2 (Mirdita *et al.,* 2022) excluding the signal sequence, showed a globular fold enriched with α-helices (Figure 1D). The globular fold of the 7th protein is well conserved among those of phylogenetically distant species ( *S. melliferum* , *S. eriocheiris* , and *S. chrysopicola* ) (Figure 1A and E). Altogether, we predict that the 7th protein is a lipoprotein that takes the globular fold on the extracellular side of the membrane. To predict the lipid modifications of the 7th protein, we examined the distribution of genes encoding three key enzymes responsible for the maturation of bacterial lipoproteins: Lgt, which adds a diacylglycerol group on the cysteine residue in the lipobox of a prolipoprotein; Lsp, which cleaves off the signal sequence of the prolipoprotein; and Lnt, acylating the amino group of the N-terminus of the prolipoprotein to mature up to the lipoprotein (Kovacs-Simon *et al.,* 2011; Buddelmeijer 2015). Lgt and Lsp were conserved in *Spiroplasma* , whereas Lnt was absent as well as other Mollicutes species (Figure 1A) (Jan *et al.,* 1995; Serebryakova *et al.,* 2011; Buddelmeijer 2015), suggesting that the 7th protein undergoes the diacylglycerol modification and the signal sequence cleavage. Regarding *N* -acylation, further studies are needed for conclusion because many lipoproteins are reported for *N* -acylation in Mollicutes species lacking the Lnt gene (Serebryakova *et al.,* 2011), including *S. melliferum* (Le Hénaff and Fontenelle 2000).

In this study, we predicted that the 7th protein is a lipoprotein whose gene is encoded downstream of *mreB3* in the Citri clade. Bacterial genes encoded closely in the same locus are often functionally related (Cohen *et al.,* 2019; Toyonaga *et al.,* 2021; Megrian *et al.,* 2022; Yamamoto *et al.,* 2023). Our analyses suggest that the 7th protein localizes to the extracellular side, whereas MreBs localize to the cells (Kürner *et al.,* 2005; Trachtenberg *et al.,* 2008; Liu *et al.,* 2017; Sasajima *et al.,* 2022). These suggest that the 7th protein and MreBs do not interact with each other for their functions. However, the position of the *7th gene* downstream of *mreB3* was conserved in every Citri clade species. Possibly this protein gives positive effects on Citri clade's survival through swimming, because Mollicutes genomes code for limited number of genes (Andersson and Kurland 1998). We also found that the 7th protein was conserved in the Citri clade species but not in the Apis clade (Figure 1A), suggesting differences in survival strategies between these clades.

## Methods

Sequences of the 7th protein were obtained by a BLAST search as the reference for *S. eriocheiris* (WP_047791952.1) with an E-value threshold of 0.05, on August 22, 2022. Sequences that were duplicated and misannotated were excluded from the sequence set for analysis (WP_004028916.1, CAK98229.1, and WP_252157057.1). Thus, the sequence set contained 14 amino acid sequences of the 7th protein (Extended Data 1). A phylogenetic tree of 16S rRNA sequences was constructed using MEGA-X, as previously described *(Kumar et al., 2018; Takahashi et al., 2020)* . The sequence of the 7th protein from *Spiroplasma* endosymbiont of *Phyllotreta cruciferae* , whose 16S rRNA sequence was not reported, was excluded from the phylogenetic and gene organization analyses. Signal peptide regions were predicted using Phobius *(Käll et al., 2004)* . The conservation level of each amino acid residue was evaluated by multiple alignment of the 7th protein sequence using Clustal Omega *(Sievers and Higgins 2018)* . The amino acid conservation of the lipobox is further evaluated by WebLogo *(Crooks et al., 2004)* . The structures of the 7th proteins were predicted from their full-length sequences using the CoLab version of AlphaFold2 *(Mirdita et al., 2022)* , and structural visualizations and removal of the signal sequence were performed using UCSF Chimera ver. 1.13.1 *(Meng et al., 2006)* .

## Extended Data

Description: The accession IDs used in this study and the summary of the analyses are shown in an Excel file containing two sets of extended data. Extended Data 1: Accession IDs and the source species of 7th proteins obtained by the BLAST search and their predicted lengths of signal sequences. Extended Data 2: Accession IDs and the source species of the genome or contig DNA sequences refer to gene orders from mreB2 to mreB5. Resource Type: Dataset. DOI: 10.22002/ba1pp-vk861

Description: PDB file predicted by AlphaFold2. Resource Type: Dataset. DOI: 10.22002/zx8y9-4b517
